# The Myth of the Middle Class Squeeze: Employment and Income by Class in Six Western Countries, 1980–2020

**DOI:** 10.1177/00104140241271166

**Published:** 2024-08-08

**Authors:** Jad Moawad, Daniel Oesch

**Affiliations:** 1 Institute for New Economic Thinking & Nuffield College, 6396 University of Oxford, Oxford, UK; 2 Centre LIVES, 27213 University of Lausanne, Lausanne, Switzerland

**Keywords:** social class, income, labor market, occupations, economic growth

## Abstract

The public debate portrays the middle class as the big losers in recent decades, while people above and below seemingly fared better in terms of employment and income growth. This narrative is both conceptually and empirically flawed. Based on the Luxembourg Income Study 1980–2020, we show for France, Germany, Poland, Spain, the UK, and the US that middle-class employment expanded, while the working class shrank. The middle class also experienced consistently larger income gains than the working class over the past four decades. The disposable real incomes of working-class households in France, Germany or the US grew by less than half a percent per year, compared to 1% or more for the middle class. Cohort analysis also shows that the promise of doing better than one’s parents held for the middle class, but vanished for the working class.

## Introduction

Since the 1980s, the growth of median incomes has slowed down across the Western world, stagnating for long periods in the United States, Germany, and France (Nolan, 2020). Income stagnation means stalled living standards for successive cohorts and has been seen as leading to the erosion of the middle class. Prominent economists and sociologists have thus argued that the middle class in Western societies is under threat (Vaughan-Whitehead, 2020), declining (Gebel, 2016; Pressman, 2007), squeezed (Autor & Dorn, 2013a; Jaimovich, 2020) and breaking apart (Chauvel et al., 2021). Based on the finding of decreasing employment and income shares of middle-income groups, this narrative has also been echoed by international organizations (OECD, 2015, 2019) and, very forcefully, by the media.^1^

Our paper challenges this narrative on both conceptual and empirical grounds. Conceptually, this literature relies on income-based definitions of the middle class that consider everyone but the poor and the wealthy as middle class (e.g., OECD, 2019; Vaughan-Whitehead, 2020). These definitions completely ignore the working class. Yet the idea that “middle-class living standards begin when poverty ends” (Ravallion, 2010, p. 446) is at odds with the history of 20th century industrial societies, which were dominated by large working classes (Cherlin, 2014).

If the working class is properly taken into account with a class scheme rooted in the occupational structure, the thesis of a squeezed middle class also becomes shaky on empirical grounds. It is undisputed that the top 1% has done very well in recent decades (Piketty, 2014; Saez & Zucman, 2020; but see Auten & Splinter, 2024). However, the main losers over the same period were not occupations belonging to the middle class or middle-income groups. Instead, it was the jobs and incomes of the workers laboring below the middle class that came under pressure: the skilled and, above all, low-skilled working class. Their labor market perspectives deteriorated sharply in the wake of skill-biased technological change, globalization, and the neoliberal turn in politics.

Our paper provides an empirical analysis of the squeezed middle-class thesis. We examine how different social classes have fared over the past four decades in terms of employment and income in six major Western countries: France, Germany, Poland, Spain, the United Kingdom, and the United States. To test the argument that the working class has lost out more broadly in recent decades, we provide additional evidence for six small and affluent European countries.

Our analysis is based on the best available comparative microdata set, the Luxembourg Income Study (LIS), which combines several dozen country surveys such as the Current Population Survey for the US and the Socio-Economic Panel for Germany. Focusing on the household level as the crucial locus of people’s life chances, we show how the working-age population has fared over the past decades in terms of household disposable income and household labor income.

Our paper makes four contributions to the literature on inclusive growth, an economic scenario in which incomes increase equally for all social classes (Baccaro et al., 2022; Nolan, 2018; Parolin & Gornick, 2021). Our first contribution is conceptual and delves into class theory. We argue that most income-based definitions of the middle class do not adequately capture the class hierarchy in Western societies. Instead, we return to occupations as the building blocks of the class structure and propose an occupation-based class indicator that reflects salient differences in the labor market hierarchy of affluent societies. By distinguishing the low-skilled from the skilled working class and the middle class from the upper-middle class, we obtain a measure of class that is both meaningful to laypeople and easy to implement in international surveys.

Second, we show that the evolution of jobs and incomes is usefully analyzed in terms of social class as the economic trajectories of classes have diverged dramatically. Since the 1980s, both the skilled and low-skilled working class have lost out in terms of employment and incomes, while the middle and especially the upper-middle class have fared much better.

Third, we take advantage of our comparative design and contrast the income trajectories of different classes across affluent countries, going beyond the single-country studies on the class-income nexus (e.g., Wodtke, 2016; Zhou & Wodtke, 2019). While our comparison reveals systematic parallels – the income hierarchy of social classes looks very similar across Western countries – it also points to notable differences. Since the early 1980s, working-class households in France, Germany and the United States have fared much worse in terms of income growth than those in Spain, the UK and especially Poland.

Fourth, our analysis shows how the diverging class fortunes played out over time for different birth cohorts. Throughout the postwar decades, each successive generation had higher incomes than the previous one. This mechanism of rising real incomes broke down in the 1980s in Germany and the United States for the working class, but not for the middle class.

In what follows, our paper first discusses the decoupling of productivity growth and income growth, before arguing that the commonly used income-based definitions provide ambiguous measures of the middle class. We replace them with an occupation-based class indicator and discuss the reasons why the working class has lost ground in recent decades. The results section shows how employment has evolved in different classes, and compares annual changes in real household disposable income across classes, cohorts, and countries. The conclusion discusses the implications of the decline of the working class for the rise of right-wing populist parties.

## Theoretical Background

### The Decoupling of Productivity and Income

Over the past two decades, absolute changes in incomes have received far less attention in political economy than relative changes in incomes as captured by indicators of inequality. Yet while the meaning of the Gini index remains obscure to most people, workers intuitively understand what annual changes in their incomes mean for their lives.

Historically, the evolution of labor income has been driven by productivity growth. For lack of a better measure, productivity is often approximated by GDP per capita and shows a continuous slowdown in the Western world. Averaged across France, Germany, Spain, the UK, and the US, productivity growth fell from an average annual increase of 3% in the 1970s to 2.5% in the 1980s and 2% in the 1990s, before leveling off at 1% per year between 2000 and 2020 (OECD statistics).

The key question is whether productivity growth translates into higher labor incomes. In the three decades after the Second World War, wages rose in line with productivity for the vast majority of Western workers (Iversen & Soskice, 2019; Piketty, 2014). Indeed, annual wage increases were the central mechanism for translating economy-wide productivity gains into broad-based improvements in living standards (Baccaro et al., 2022). The 1980s was the watershed decade in which the Keynesian class compromise based on full employment and collective bargaining fell apart – and when the link between productivity growth and workers’ earnings became loosened (Piketty, 2014; Stansbury & Summers, 2018). As a disproportionate share of labor income went to those at the top of the wage scale, ordinary workers were left empty-handed.

While income from labor is the most important source of income for most households, all affluent countries redistribute some income through taxes and transfers. Household *disposable* income – after taxes and transfers – therefore tends to be less unequally distributed than market income (Gornick & Smeeding, 2018). Despite the rhetoric of welfare retrenchment, social spending increased in most affluent countries between the 1980s and 2000s (Marx & Van Rie, 2014). Governments thus continued to rebalance income distribution through taxes and transfers, but were unable – or unwilling – to stem the tide of increasingly inegalitarian markets (Caminada et al., 2019, p. 5; Dallinger, 2013; Kenworthy & Pontusson, 2005). As a result, household disposable income also tended to become more unequally distributed (Piketty, 2014). Since the 1980s, median household disposable income has lagged behind average household income in most Western countries, because households at the top have captured a disproportionate share of total national income (Nolan, 2018; Nolan & Thewissen, 2018; Nolan & Weisstanner, 2022).

### The Crux of Defining the Middle Class as a Middle-Income Group

These shifts in income have been interpreted as squeezing the middle class (OECD, 2019). However, few concepts are as fuzzy and difficult to define as that of the middle class (Cherlin, 2014). This difficulty has been exacerbated by recent studies on the decline of the middle class, which define the middle class as large middle-income groups (e.g., Gebel, 2016; Grabka & Frick, 2009; Pressman, 2007; Ravallion, 2010). In particular, two income-based definitions of the middle class have proved influential.

The first definition, originally endorsed by the OECD (2015), considers the middle 60% – households between the 20th and 80th income percentiles – as middle class (see also Dallinger, 2013). Based on this definition, the income share accruing to the middle three quintiles declined by a few percentage points between the mid-1990s and the late 2000s in the United States and most European countries, especially in France and Germany, but not in Eastern European countries or the Netherlands (OECD, 2015, p. 33).

A second definition – favored by the ILO (Vaughan-Whitehead et al., 2016) – includes all households with more than 60% and less than 200% of the median income in the middle class (see also Atkinson & Brandolini, 2013). Based on this definition, the middle-income group grew in most European countries in the early 2000s, but declined after the Great Recession – with more favorable employment and income trends in Eastern Europe than in France, Germany, and Southern Europe (Vaughan-Whitehead et al., 2016).

More recently, the OECD (2019) used a similar definition of the middle class, including all households with incomes above 75% and below 200% of the median. Based on this definition, the OECD-average share of people in middle-income households fell from 64 to 61% between the 1980s and 2010s, with larger declines in Germany and the US than in France and the UK (OECD, 2019, p. 49).

These results have been interpreted as showing a decline in the middle class. However, we argue that the income-based measures provide ambiguous indicators of the middle class for several reasons. To begin with, they result in a very large and heterogeneous middle class. In the first definition, it encompasses, by construction, the middle 60% of households. In the second definition covering percentiles 75 to 200, it comprises between 65 (Germany, France) and 70% (Netherlands, Norway) of all households in Western Europe (OECD, 2019, p. 43).

Moreover, these definitions set a very low threshold for belonging to the middle class. Focusing on income percentiles 20 to 80 leaves out only the bottom quintile. However, almost 20% of the working-age population in Western Europe receives unemployment, disability, sickness, or social assistance benefits (OECD, 2003, p. 175). Thus, this definition includes all households in the middle class except those living on social benefits. For the US, this means that people either struggle to afford food and are eligible for food assistance (SNAP) – or they are middle class and lumped together with households earning five times as much.

A similar problem arises when the lower threshold for inclusion in the middle class is defined as 75% – or, a fortiori, 60% – of the median wage. In countries as diverse as Chile, France, Korea or New Zealand, the minimum wage in 2020 exceeded 60% of the median wage (OECD statistics). This means that whoever lives in a one-person household and earns close to the minimum wage is part of the middle class – yet minimum wages are typically paid for the most menial jobs in fast-food restaurants or cleaning services.

By including in the middle class whoever holds a job and is not poor, these definitions are ahistorical because they ignore the working class – the majority class for much of the 20th century in Western industrial countries (Todd, 2014). Historically, the middle class included a small category of non-manual employees such as lawyers and merchants, doctors and priests, civil servants and teachers who were situated below the tiny powerful elite of factory owners, entrepreneurs and landowners, but above the large working class laboring in manual jobs as farmworkers, construction workers, factory workers, or domestic aides (Hobsbawm, 1999; Kocka, 1995).

Consistent with its majority status, the term “working class” appeared more frequently than “middle class” in English-language books for most of the 20th century, between 1907 and 1990 (Oesch, 2023). Even today, the distinction between the middle and working class remains entrenched in the everyday lexicon – between workers and employees, manual and non-manual work, blue-collar and white-collar jobs –, and many people still consider themselves as working class. According to the International Social Survey Programme, 36% of Americans and 40% of Britons and Spaniards saw themselves in 2009 as working class, but only 1% as upper class (Oesch & Vigna, 2023). The skewed size of the (large) working class at the bottom and the (small) upper class at the top dispels the misconception that the middle class is concentrated in the middle of the income structure. In most Western countries, carpenters and mechanics, bricklayers and truck drivers earn wages close to the national median. Historically, these blue-collar occupations formed the backbone of working-class unions, and few sociologists would consider them to represent the middle class.

### Grasping the Middle Class with an Occupation-Based Concept

If these definitions of the middle class simply reflect large middle-income groups (Gornick and Jäntti 2013), how else can the middle class be conceptualized? We follow the dominant tradition in stratification research that considers occupations to be the cornerstones of contemporary labor markets and the resulting class system (DiPrete et al., 2017; Weeden & Grusky, 2005). Besides income levels, occupations share other crucial properties such as training requirements, working conditions and geographical location – suffice to compare the specific working conditions and workplaces of farmers and miners, cashiers and cooks.

We argue that the class hierarchy can be seen as arising from the technical division of labor that is rooted in the occupational structure. Workers in different occupations control different amounts of productive resources which, in turn, place them into asymmetrical social relations to each other (Wodtke, 2016; Wright, 1997). Typical examples are the contrasts between doctors and nursing aides, managers and secretaries, engineers and machine operators.

In Western societies, close to 90% of labor market participants do not own their businesses, but work as employees. For most people, the key productive resource is therefore not capital, but skills (Wright, 1997, see also Erikson & Goldthorpe, 1992, p. 32). The more skills an occupation requires, the more difficult the workers are to replace, the more bargaining power they wield, and the more advantageous their work contracts are (Goldthorpe, 2000; Le Grand & Tåhlin, 2013). For empirical research, therefore, an occupation’s productive resources – and thus its position in the class hierarchy – can be proxied by its skill requirements.

On this basis, we propose to distinguish four social classes that comprise occupations with similar levels of skill requirements: an upper and upper-middle class (short: upper-middle class), a middle class, a skilled working class, and a low-skilled working class. We separate the upper-middle class of professionals and managers from the core of the middle class which includes semi-professionals, associate managers, and technicians. While access to the professions and many positions in management requires the equivalent of a university degree, shorter post-secondary degrees are typically sufficient to become an associate professional or technician. A similar logic applies to the division within the working class. Skilled working-class occupations normally require a few years of upper-secondary education – often in the form of vocational training –, whereas low-skilled working-class occupations are entry-level jobs that can be learned in a few months of on-the-job training.

Schematically, a four-fold occupational hierarchy exists in many large organizations, be it in manufacturing (engineers, technicians, welders and assemblers), hospitality (general managers, accountants, cooks and dishwashers) or health care (doctors, nurses, nursing aides and cleaners). As we will discuss in the measurement section, it also translates easily into the two most widely used class schemes in sociology, EGP and ESeC, both associated with Erikson and Goldthorpe (1992).

### The Squeezed Working Class

Once the working class is brought back from oblivion, it becomes easier to argue that the middle-class squeeze is really a working-class squeeze. It is not middle-class occupations nor middle-income groups that fared worst in terms of employment and income growth, but working-class occupations that are set in the bottom half of the income distribution.

The decline of the working class began in the 1970s, which marked both the peak and end of the golden age of industrial capitalism. Under the impact of skill-biased technological change and globalization, the working class began to shrink as labor demand dried up for welders and assemblers, typists and switchboard operators. In addition to technological change and offshoring, the working class was further squeezed by the neoliberal turn in political economy and the return of mass unemployment in the 1980s (Eichengreen, 2008; Hall, 2020).

Over the same period, the middle class was anything but in decline. The ongoing race between technology and education – between skill-biased technological change and educational expansion – led to a steady increase in its ranks across the Western world. In Britain, the share of the labor force employed in working-class occupations fell by a third between 1951 and 2011. In parallel, the middle class – defined as lower and higher managerial and professional occupations – increased its share from ten to over 30% (Bukodi & Goldthorpe, 2018, p. 36). Similarly, in Germany and the United States over the course of the 20th century, each successive birth cohort has been less likely to work in an unskilled manual job and more likely to hold a middle-class job (Breen and Müller 2020, p. 252). In the United States, between 1980 and 2015, employment dropped among production workers, laborers, and office clerks, but expanded rapidly among technicians, professionals, and managers (Autor, 2020, p. 114). The simultaneous loss of working-class jobs and gain of middle-class jobs has also been documented for France between 1982 and 2018 (Goux & Maurin, 2019) and for Germany, Spain, and the UK between 1992 and 2015 (Oesch & Piccitto, 2019).

As working-class jobs became harder to find, trade union membership declined and the working class lost bargaining power. Between 1980 and 2020, union density fell by half in France, Germany, the UK, and the US (OECD statistics). Over the same 40 years, only a few large Western countries, such as France and Spain, maintained stable collective bargaining coverage above 80%. In the US, however, it fell from 25 to 12%, and in Germany and the UK, it dropped from over 80 to 50% and 25% of the workforce, respectively (OECD statistics). The weaker bargaining power of the working class is reflected in the reduction of industrial conflict, with strikes falling to historically low levels in OECD countries in the 2000s and 2010s (Van der Velden, 2007; Vandaele, 2016).

The working class did not only lose economic, but also political power as social-democratic and Labour parties – its traditional allies – moved to the center. Faced with a shrinking base of working-class voters, the working class ceased to be the top priority of the parties of the left. Instead, these parties began to court the salaried middle class (Hall et al., 2023).

In the context of weaker labor demand, neoliberal economic policies, and eroding bargaining power, the working class struggled to secure its share of economic growth. Income differences between social classes increased massively in the US between the 1980s and 2000s (Wodtke, 2016, see also Ikeler & Limonic, 2018) and also widened in Europe after the Great Recession (Albertini et al., 2020; Moawad, 2023).

Based on these elements, we argue that the working class has systematically fared worse than the middle class in large Western countries since the 1980s. For this argument to hold, our analysis must show that different classes have diverged in terms of employment and, crucially, income growth. In addition, we document how the diverging class fortunes have played out over time for different birth cohorts. During the golden age of the post-war decades, the Silent Generation (1926–45) and the Baby Boomers (1946–65) enjoyed rising material standards across the Western world as real incomes increased for each successive cohort. This upward trajectory has slowed down and may even have stalled for Generation X (born 1966–1980), particularly in France (Chauvel & Schroder, 2014, 2015) and the UK (Anderson, 2022). However, the economic slowdown experienced by Generation X should have left a much deeper mark on the incomes of the working class than on those of the middle class.

## Data and Methods

### Data, Sample and Income Measure

Our analysis uses data from the Luxembourg Income Study (LIS) and focuses on six large Western countries: France, Germany, Poland, Spain, the United Kingdom and the United States. Our selection is based on three criteria. The first criterion is the availability of comparable occupational data over several decades which excludes Italy. The second criterion is population size and we include five of the six most populous Western European countries (excluding Italy) in addition to the United States. A third criterion relates to institutional diversity in terms of markets and states – welfare capitalism – and provides us with examples of the liberal Anglo-Saxon regime (the UK and US), the conservative continental regime (France and Germany), the conservative Mediterranean regime (Spain), and the post-socialist regime (Poland) (Esping-Andersen, 1999). Finally, these six countries were major players in the world economy during the period under study. At our starting point in 1980, they together accounted for almost half of world industrial production (Christian, 2004, p. 408). However, we expect our argument about the squeezed working class to hold more broadly, and we also present results for six small and affluent European countries for which we have consistent occupational data over several decades: Austria, Denmark, Finland, Ireland, the Netherlands, and Switzerland.

The LIS database constitutes a unique source of cross-nationally comparable income data. For each of our selected countries, it assembles more than a dozen annual surveys, such as the Current Population Survey for the US, the Socio-Economic Panel for Germany, the Household Budget Surveys for France, Poland, and Spain, or the Family Resources Survey for the UK.

Our central argument is that the labor market prospects of the working class were hampered by a historical trend that started in the 1980s and continued for the next three decades. We take 2018 as our endpoint, because that year is covered by a LIS survey in all six countries. We then go back four decades from this benchmark, selecting the closest module with consistent information on occupations and employment status. Thus, our starting point is 1978 for the US, 1980 for Spain, 1984 for France and Germany, 1998 for the UK and 1999 for Poland. For some countries, earlier modules exist, but changes in occupational variables make the consistent comparison with later modules all but impossible. Our analysis, therefore, covers longer periods for the US (1978–2018), Spain (1980–2018), France and Germany (1984–2018)^2^ than for the UK (1998–2018) and Poland (1999–2018).

Most families pool their resources between household members, especially for housing and food. Household income is therefore a better measure of life chances than individual income. Our dependent variable is household disposable income, adjusted for both inflation (using LIS consumer price indices) and household size (using the LIS equivalence scale: the square root of the number of household members). Household disposable income includes labor and capital income as well as government transfers, but deduces taxes. As our interest is in how different classes have fared over time within a given country, we avoid exchange rate fluctuations and do not convert incomes into U.S. dollars using purchasing power parities (see Atkinson et al., 2017).

We analyze the household disposable income of the working-age population, removing households where the main earner was younger than 25 (and thus possibly still in education or training) or older than 60 (and thus possibly retired). Following the LIS convention (Caminada et al., 2019), we also exclude the small share of households that do not report any annual income. For all our analyses, we use the household weights provided with the data.

### Measuring Social Class

Our main independent variable is social class, which distinguishes occupations on the basis of the productive resources they possess. For workers, the main productive resources are the skills typically required in their occupation, in contrast to employers who also rely on capital. We measure people’s occupation at the ISCO-88 1-digit level. Although a finer distinction would be preferable, consistent information for six countries over four decades is only available at this level. Even at this level of aggregation, ISCO distinguishes four hierarchical skill levels which have strong parallels with our four-tier class hierarchy (Elias, 1997).^3^ In order to obtain a more robust measure of the skill level typically required by occupations, we additionally use information on people’s education, distinguishing between low (primary and lower secondary education), medium (upper-secondary education) and high levels (tertiary education).^4^ Finally, we separate employers and the self-employed from employees, as the former two groups own their own business and thus also have capital as a productive resource. Our four classes are then constructed based on the following logic:

(A) The *upper and upper-middle class* (hereafter upper-middle class) includes occupations set at the highest skill level, professionals and managers. To begin with, it encompasses all employers whose occupation is manager or professional, irrespective of their education. We further add all employees and self-employed working as professionals (ISCO 2) with a university degree, as well as all employed managers (ISCO 1) with at least upper-secondary education. By contrast, the minority of managers who are self-employed (and have no employees and therefore only manage themselves) or have only post-compulsory education (and are likely to manage small outlets such as shops or bars) are shifted downwards to the middle class.
(B) The core of the *middle class* consists of associate professionals, technicians and associate managers (ISCO 3). Their occupations are set at the second highest skill level and typically require some form of post-secondary education, but not necessarily a university degree. We also include professionals (ISCO 2) with no tertiary education (such as pre-primary teaching professionals and social workers in some countries) as well as office clerks (ISCO 4) with tertiary education (such as accounting clerks). Finally, the middle class includes all employers whose occupation is neither managerial nor professional (ISCO 3–8) and who do not work in an elementary occupation (ISCO 9). This means that employers in agriculture, hospitality or construction are part of our definition of the middle class.
(C) The *skilled working class* includes employees and the self-employed in occupations set at the third highest skill level, requiring a few years of upper-secondary education, typically in the form of vocational training (Elias, 1997). It comprises clerical workers without tertiary education such as secretaries (ISCO 4), all service and sales workers (ISCO 5) and all craft workers (ISCO 7). It also includes self-employed farmers (ISCO 6) who, not having any employees, are likely to be small farmers. This class is completed by two minor groups: the armed forces (ISCO 10) and self-employed machine operators and assemblers (ISCO 8) – a very rare combination.
(D) The *low-skilled working class* includes workers in occupations with the lowest skill level – entry-level jobs that do not require upper-secondary education. In addition to laborers in elementary occupations (ISCO 9), irrespective of their employment status, this class includes agricultural workers (ISCO 6) and plant and machine operators and assemblers (ISCO 8). The coding of our class variable is presented in Table A.1 in Appendix A.

Some households are composed of individuals – typically partners – whose occupations are set in different social classes. To ensure that we assign household income to the correct household class, we exclude these cross-class households from the analysis. This reduces our country samples by 15%, but still leaves us with large samples averaging 12,382 observations per survey year in Germany, 44,373 in France, 46,294 in Poland, 14,808 in Spain, 22,214 in the UK and 77,688 in the US. We provide a robustness test that keeps all households and attributes to each household the *dominant* (i.e., highest) class of its members (Erikson, 1984). The descriptive statistics are presented in Table A.2 in Appendix A. The replication materials and R codes of all our analysis are freely available at Moawad and Oesch (2024).

Conceptually, our class measure shares the same starting point as EGP and ESeC, the two most widely used class measures in empirical research (Erikson & Goldthorpe, 1992; Rose & Harrison, 2010): the more specialized skills an occupation requires, the more advantageous is the typical class setting offered by the employer (Tåhlin, 2007). However, EGP and ESeC are much more demanding in terms of data. In addition to occupation and employment status, they also require information on the number of employees and, crucially, on supervisory status. These two variables are typically missing in LIS, making it impossible to consistently construct these class measures over several decades.

However, most occupation-based class measures end up being strongly correlated in empirical analyses (Bihagen & Lambert, 2018; Lambert & Bihagen, 2014) and it is therefore possible to pinpoint how our four classes translate into ESeC and EGP (see Table A.3 in Appendix A). The upper salariat (or higher-grade service class) roughly corresponds to our upper-middle class, and the lower salariat (or lower-grade service class) to our middle class. At the bottom end, semi- and unskilled workers (along with large proportions of lower-grade white-collar workers) make up the low-skilled working class, while skilled white- and blue-collar workers form the skilled working class.

### Method

Our analytical strategy follows the descriptive turn in the social sciences (Piketty, 2014) and shows the mean annual change in household incomes by social class in percent. An example illustrates our calculation: The equalized, inflation-corrected disposable income of a low-skilled working-class household in the US was $30,000 in 1978 and $31,200 in 2018. This represents an income gain of 4.0% [(31,200–30,000)/30,000)] over 40 years, which corresponds to a mean increase of 0.1% (4.0/39) per year. Our results section presents these mean annual changes in real household disposable income, adjusted for household size.

When calculating the annual income growth for a given class over a given period, the actual range of years considered can make a big difference due to business cycle fluctuations. Data availability makes it difficult to use the same starting year for all countries. However, even a common starting year would not correct for differences in business cycles across countries (Nolan, 2020). Instead, we address this issue by also showing how annual income varied across countries from decade to decade as well as for a common period of twenty years, 1998–2018.

In addition, we examine how historical trends played out for different birth cohorts. We do so by distinguishing three sociologically significant birth cohorts that began their work careers in different historical contexts: the Silent Generation, born 1926–1945, the Baby Boomers, 1946–1965, and Generation X, 1966–1980 (Howe & Strauss, 1992). For the cohort analysis, we restrict the sample to individuals between the ages of 35 and 50 to compare the same age range for each cohort and thus avoid out-of-sample predictions (our data contain no observations below age 35 in the Silent Generations or above age 50 in Generation X).

For these cohort analyses, we estimate the following equation:

(1) $${\gamma \left(\log\;\_income\right)}_{it}=\beta 0+\beta 1{Class}_{it}+\beta 2{Cohort}_{t}+\beta 3{Class}_{it}*{Cohort}_{t}+\beta 4{Age}_{it}+\varepsilon_{it}$$

The interaction effect between class and cohort allows us to show how classes’ income trajectories varied across cohorts, controlling for age in years. It tells us how household disposable income evolved over the life course for workers in the same social class who were born in different periods of the 20th century. This answers the question of whether working-class children born in the 1970s were worse off than their working-class parents and grandparents. Again, our goal is descriptive and does not aim to causally disentangle cohort, period and age effects.

## Findings

### Change in Employment

**Figure 1.**
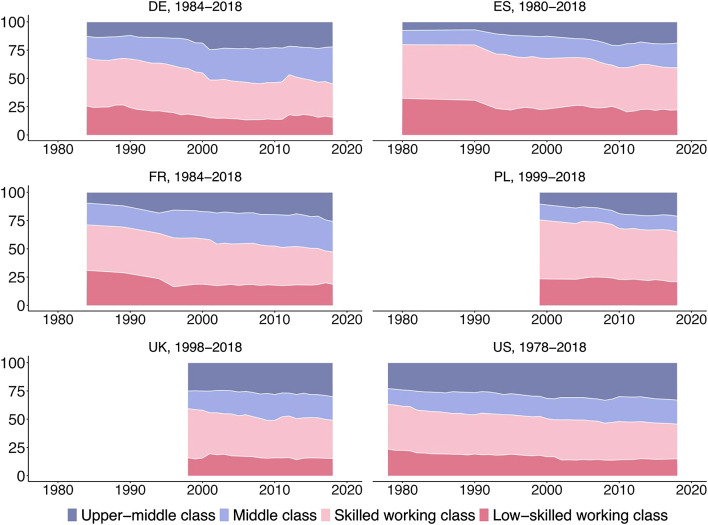
The class composition of the working-age population over time (in %).

Before analyzing the evolution in incomes, we need to document that labor demand has been biased against the working class. For this reason, Figure 1 shows how the class composition of the economically active population aged 25–60 has evolved over the last four decades. In the 1980s and 1990s, the two working classes clearly outnumbered the two middle classes in all six countries. With more than a third of the workforce, the skilled working class was initially the largest class in all countries. Three to four decades later, the class composition looks very different, with the middle class and upper-middle classes together accounting for roughly the same employment share as the skilled and low-skilled working classes in Germany, France, the UK, and the US – but not in Poland and Spain where the working classes remain larger.

These findings do not seem to be driven by differential selection into employment over time. In the five countries for which the OECD provides long-term labor market data (all but Poland), the share of households headed by an adult aged 25 to 60 in which *no member* was in paid employment – because of unemployment or economic inactivity – decreased over time: from an average of 27% in the 1980s to 22% in the 1990s, further diminishing to 19% in the 2000s and then to 18% in the 2010s.

**Figure 2.**
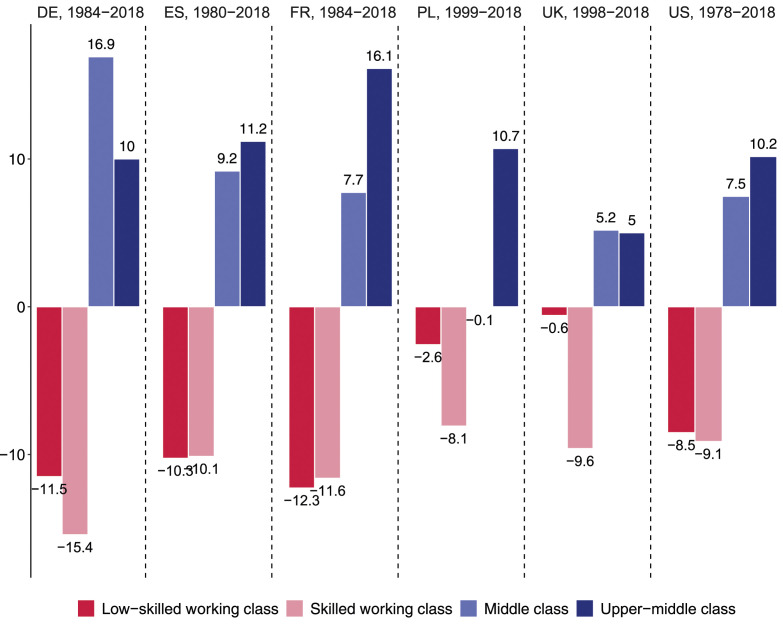
Change in the employment share of different classes (in percentage points).

The employment shifts observed for the different classes are summarized in Figure 2. The transformation of the class structure followed a similar pattern across the six countries studied. In France, Germany, Spain and the US, the employment share of the skilled and low-skilled working class fell by about the same extent – between 8 and 15 percentage points each –, while the employment share of the middle and upper-middle class increased by the same amount, with slightly stronger growth for the upper-middle than the middle class. For these four countries, our results paint a clear picture of occupational upgrading as employment expanded in the upper-middle and middle class at the expense of the two working-class segments.

The picture is somewhat different in Poland and the UK where job growth was concentrated in the upper-middle class and job losses among the skilled working class, while the employment share of the low-skilled working class remained almost stable. The result is a slightly polarizing class structure that sets these employment trajectories apart from that of other Western European countries.

### Change in Household Disposable Income

**Figure 3.**
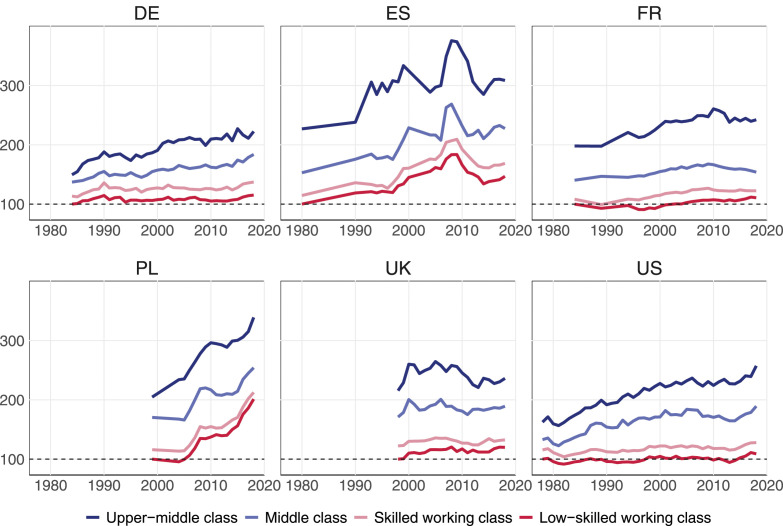
Evolution of indexed real household disposable income by class over time.

While the middle class has not been squeezed in terms of employment, it may still have lost out in terms of income. Figure 3 therefore shows how real household disposable income evolved for different classes over the last few decades. For ease of interpretation, we set the income of the low-skilled working class at 100 in the first observed year within each country and express all other incomes relative to this reference value. These results show the expected income hierarchy between social classes for each country: Upper-middle-class households earn, on average, the highest incomes and low-skilled working-class households the lowest, with the middle class and the skilled working class in-between.

Comparing the income evolution of the working class between countries, three patterns emerge. In France, Germany and the United States, both the skilled and unskilled working class treaded water over the past three decades, as their inflation-adjusted household incomes stagnated between the early 1980s and 2018. Over the same period, middle-class and especially upper-middle-class households continued on a path of rising incomes. The result is a widening of the income gap between classes in these three countries.

In the UK, not only the middle and upper-middle class, but also the working classes experienced some modest income growth during the boom period of the 2000s until the Great Recession. Finally, in Poland and Spain, all households experienced substantial income growth. In Poland, incomes rose sharply throughout the study period (1999–2018), whereas Spain’s period of strong income growth lasted only from the mid-1990–2008, when the housing bubble burst.

**Figure 4.**
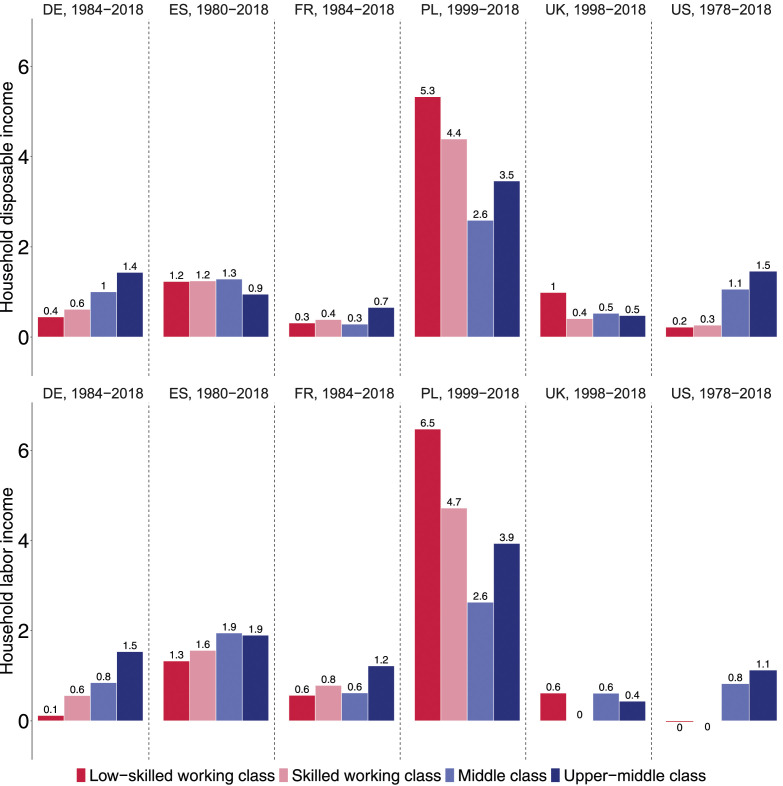
Annual mean change in household disposable income (upper panel) and household labor income (lower panel), in %.

The income evolution of different classes becomes more tangible when presented on an annual basis. This is done in our key Figure 4, which confirms that class fortunes diverged most strongly in Germany and the United States. In Germany, the real disposable income of the low-skilled working class increased by only 0.4% per year on average. The skilled working class also fared poorly with weak income gains of 0.6% per year, followed by the middle class with annual gains of 1%. By contrast, the upper-middle class experienced substantial annual income gains of 1.4%. Nearly the same pattern can be observed for the US where the household disposable incomes of the low-skilled and skilled working class have been almost stagnant over the last four decades, increasing annually by only 0.2 and 0.3% respectively between 1978 and 2018. In contrast, the middle and upper-middle classes experienced robust annual gains of 1.1 and 1.5% respectively. In the US, the middle and upper-middle class thus earned, on average, 1% more per year than did the low-skilled working class over the past four decades. Compounded over 40 years, this amounts to a widening income gap of 49%.

In France, we observe similar orders of magnitude at the bottom end of the class structure, with annual income growth of 0.3% for the low-skilled working class and 0.4% for the skilled working class. However, in contrast to Germany and the US, the middle class in France did not do any better (0.3%). Only the upper-middle class saw a slightly faster increase in household incomes (0.7%). Class disparities were inversed in Spain and the UK. In Spain, the two working-class segments and the middle class saw their disposable incomes grow by about 1.2–1.3% and thus slightly faster than for the upper-middle class (0.9%). In the UK, low-skilled working class households also benefitted from stronger income growth (1%) than the three other class (0.5%). Note, however, that these results only apply to *disposable* incomes and thus show the redistributive nature of taxes and transfers. If we focus instead on household *labor* income (in the bottom panel of Figure 4), class differences disappear in the UK and shift in favor of the middle and upper-middle class in Spain.

Poland is a case apart where annual incomes rose much faster than elsewhere in the early 21st century. Its middle and upper-middle classes fared much better than their counterparts in the large Western countries, with annual income gains of 2.6 and 3.5% respectively over the last two decades. Yet the rewards of Poland’s strong economic growth were not skewed toward the upper-middle class, but benefitted the low-skilled working class the most. With annual income gains of 4 and 5%, respectively, Poland’s skilled and low-skilled working-class households almost doubled their disposable income between 1999 and 2018. However, our LIS data may overstate the extent to which working-class incomes have caught up in Poland. A comparison of Polish tax records and Polish survey data suggests that surveys underestimate the evolution of income inequality and, in particular, the rise in top incomes (Bukowski & Novokmet, 2021).

In the lower panel of Figure 4, we compare increases in household disposable income with increases in household labor income, which is by far the largest source of income for most working-age households (Salverda & Haas, 2014, p. 79). As expected, the class gaps tend to be larger for labor income than for disposable income. Without some redistribution through taxes and transfers, low-skilled working-class households in Germany and the US would have fared even worse. Their labor income has risen by only 0.1% per year on average in Germany and has even been frozen in the US over the past 40 years. Similarly, in France and Spain, the income trajectories are more unequal when we focus on household labor income rather than household disposable income. These results confirm earlier findings that rising income inequality is driven by changes in market rewards rather than changes in the welfare state (Caminada et al., 2019; Marx & Van Rie, 2014).

### Differences Across Birth Cohorts

**Figure 5.**
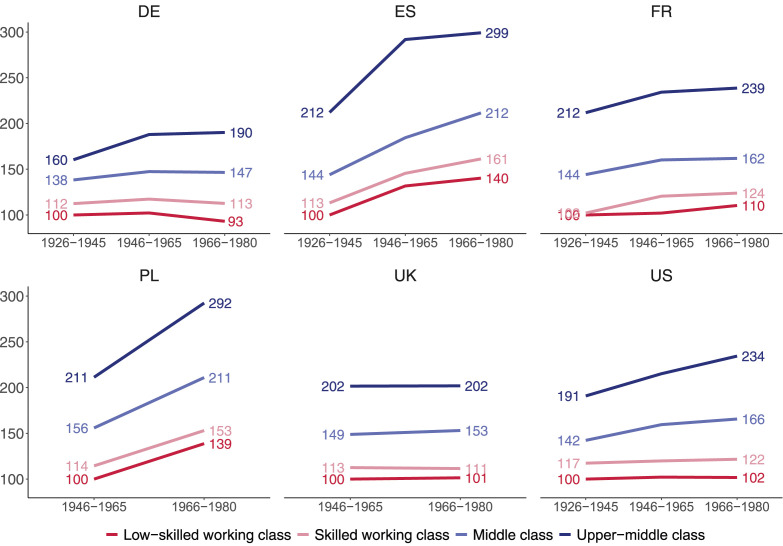
Household disposable income by class and cohort (adjusted predictions).

How did the income evolution of classes vary across birth cohorts? Figure 5 shows the predicted household disposable income by class for three cohorts. These analyses control for age (in single years) and are restricted to households headed by individuals aged 35 to 50. For ease of interpretation, we set the income of the low-skilled working class born into the Silent Generation (1926–45) at 100 and express all other incomes relative to this reference value.

When comparing the experiences of different working-class cohorts, three income trajectories can be distinguished. First, a *downward trajectory* describes the experience of Germany’s low-skilled working class. Their incomes at a given age were highest in the Silent Generation, stagnated for the Baby Boomers (1946–65) and declined for the low-skilled working class in Generation X (1966–1980). In this class, each successive generation had to settle for lower incomes than the Silent Generation whose early working lives coincided with the *Wirtschaftswunder* – Germany’s economic miracle of the post-World War II decades.

Second, a *stagnant trajectory* applies to the skilled working class in Germany as well as the low-skilled and skilled working class in the United States. In the US, in stark contrast to the middle and upper-middle class, members of the working class born into the Baby Boomer and Generation X cohorts made no income gains compared to their working-class parents and grandparents. In the US as in Germany, the living standards of working-class households had stagnated for successive generations. In the United Kingdom, there was no improvement in incomes at all for the Generation X relative to the Baby boomers in all four classes.

Third, a weak *upward trajectory* applies to France where subsequent working-class cohorts did slightly better than the working class in the Silent Generation. However, income gains were meager. A clear upward trajectory can only be observed for the working class in Poland and Spain. In Spain, working-class households of Generation X earned inflation-adjusted incomes that exceeded those of the Silent Generation by 40% for the low-skilled working class and by 48% for the skilled working class. Similarly, in Poland, the working-class incomes of Generation X exceeded those of the Baby Boomers by more than a third.

When the focus shifts to the middle and upper-middle classes, the differences between countries narrow. Middle-class disposable income grew strongly across birth cohorts in Poland and Spain, more slowly in the US, France and Germany. While the middle class fared better than the two working-class segments over successive cohorts, their income increases were, in turn, dwarfed by those of upper-middle-class households. The income trajectory of the upper-middle class tended to be steeper across cohorts than that of the other three classes. Not only in Poland and Spain, but also the US and even Germany did upper-middle class households earn substantially higher incomes over subsequent cohorts. The exceptions are France and the UK, where the income trajectories of the middle classes were parallel to those of the working classes (being flat for all of them in the UK).

## Robustness Tests

### Different Measures of Stratification

We examine the robustness of our results with several tests. To begin with, instead of excluding cross-class households, we use the class dominance method which consists of assigning households the highest class position in each household (Erikson, 1984). So if one partner is in the upper-middle class and the other partner in the skilled working class, the household is coded as being in the upper-middle class. Using this definition, we find very similar patterns of income growth by class for all six countries (see Figure A.1 in Appendix A).

**Figure 6.**
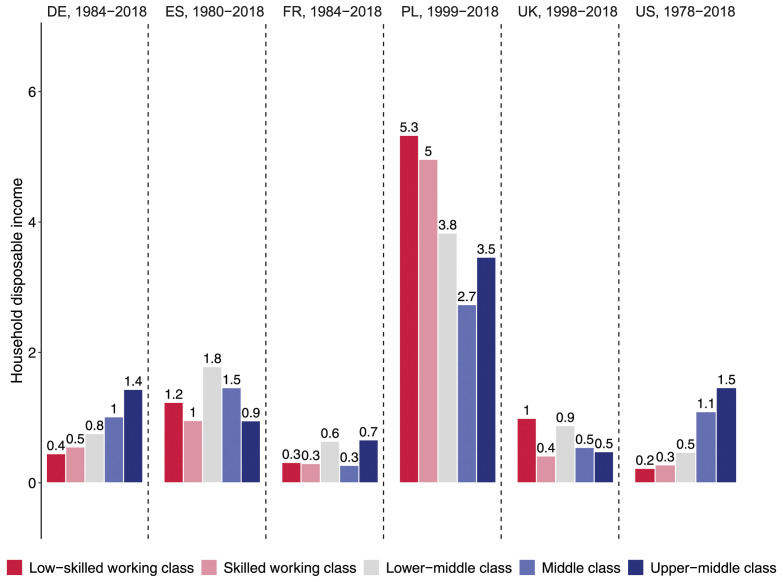
Annual mean change in household disposable income based on 5-class schema, in %.

We then resort to a more detailed 5-class measure that additionally distinguishes the *lower-middle class* which includes office clerks (ISCO-4) as well as small employers and the self-employed who are neither professionals nor managers (for the coding, see Table B.1 in Appendix B). Using this five-class measure, we still observe a strong fall in jobs for the low-skilled working class in all countries but Poland and the UK, a weaker decline in employment for the skilled working class, while the middle and upper-middle classes experienced massive job growth. The lower-middle class followed a similar employment trajectory as the working classes, with its employment share falling sharply in France and the US, and more moderately in Spain, Poland and the UK, while remaining almost stable in Germany (see Figures B.1 and B.2 in Appendix B). In terms of income, Figure 6 shows that the lower-middle class tended to do better than the working class in all countries except Poland. Yet they experienced less income growth than the middle and upper-middle class in Germany and the US, but more income growth in Poland, Spain and the US. The 5-class scheme thus leads to similar conclusions as the more parsimonious 4-class measure (see also B.3 in Appendix B).

A further concern is that our finding of a working-class squeeze is driven by a selection effect. As educational expansion and occupational upgrading allowed many working-class children to move into middle-class jobs (Breen and Müller 2020), the decreasing number of people employed in working-class occupations may have become more negatively selected. We explore this argument by dividing our analytical sample into four income quartiles. To the extent that the relative size of income quartiles is, by definition, constant over time at 25%, there is no reason why the bottom income quartile should be more negatively selected in the 2010s than in the 1980s.

When we rerun our analysis with four income quartiles instead of four social classes, we observe the same hierarchy of income gains (see Figure A.2 in Appendix A). In Germany and the US, household disposable income increased most in the fourth (top) quartile, followed by the third quartile, but grew the least in the first (bottom) quartile. A similar, albeit weaker hierarchy can also be seen in Spain. In the other three countries, the income evolution is more equal, with the bottom quartile faring better than the rest in Poland. In France and the UK, the differences in income evolution across quartiles are negligible. These results provide no evidence that our findings are driven by increasingly negative selection into the shrinking working class. Moreover, they do not support the argument that middle-income groups – quartiles 2 and 3 between percentiles 25 and 75 – lost out in terms of income relative to quartiles 1 and 4.

### Differences Over Time

In a further test, we examine whether our conclusions are unduly influenced by period differences. We therefore calculate the annual growth of household disposable income for each decade separately as well as for a common period 1998–2018 (see Figures A.3 to A.5 in Appendix A). The results for Germany show that the 1990s and 2000s in particular were lost decades of income stagnation for the middle and upper-middle classes. Over the same period, the fortunes of the two working-class categories were even worse, with substantial income losses, especially in the 1990s. Germany’s economic horizon brightened only in the 2010s, when all classes experienced income growth, with the two working-class segments benefitting disproportionately from the return to full employment and the introduction of a national minimum wage in 2015.

In the US, the middle and upper-middle classes fared better than the working classes in every single decade. The Clinton boom of the 1990s and the recovery from the Great Recession in the 2010s brought income growth for all classes. By contrast, the 1980s and 2000s saw a decline in disposable income for the two working-class segments. While the decline was sharpest in the 2000s ending with the Great Recession, the class contrast in income growth was strongest during Reagan’s reign in the 1980s.

Income growth in the UK was driven by the boom period of the late 1990s and early 2000s. With full employment and the introduction of a minimum wage in 1999, the low-skilled working class was able to make up some ground in the 2000s. However, the depth of the Great Recession in the UK meant that growth in disposable incomes remained very weak across the class structure over the 2010s – as was the case in France for most of the last forty years.

In Spain, disposable incomes grew strongly in the 1980s, 1990s and early 2000s. However, income growth came to a halt with the Great Recession and the (early) 2010s were marked by austerity and falling real incomes. Finally, Polish households enjoyed strong income growth in both the 2000s and 2010s. Yet it was only in the latter decade that growth in disposable incomes became skewed in favor of the two working-class segments.

### A Different Set of Countries

Finally, skeptical readers may wonder whether our findings of a working-class squeeze are due to cherry-picking a few large Western countries. We therefore replicate our analysis for six smaller West European countries for which LIS provides consistent information on incomes and occupations over at least two decades. In these countries as well, relative employment decreased in the low-skilled and skilled working class, but expanded in the middle and, above all, upper-middle class (see Figure A.6 in Appendix A). Two exceptions are Denmark and Ireland where the employment share of the middle class also fell, due to a decline among the self-employed and small employers.

**Figure 7.**
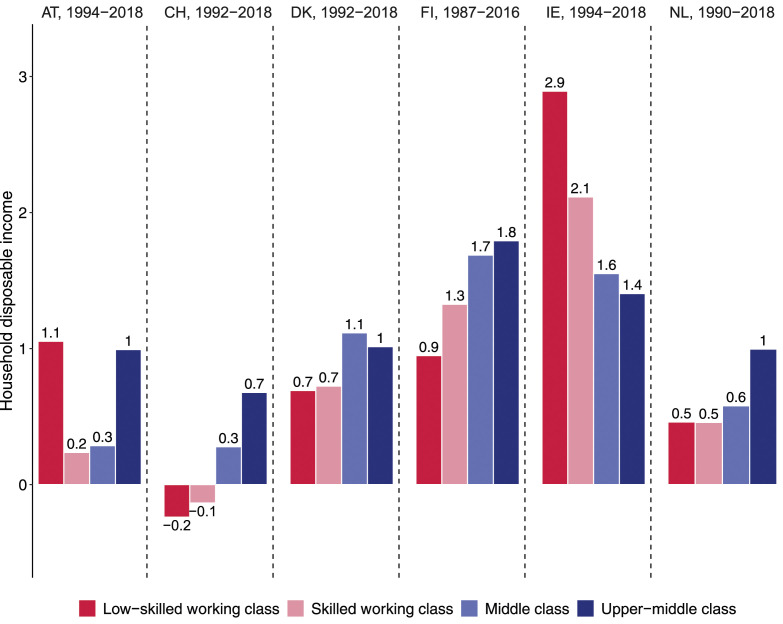
Annual change in household disposable income in small European countries, in %.

In terms of income evolution, Figure 7 shows the same skewed class pattern for income growth in Denmark, Finland, the Netherlands, and Switzerland as observed for Germany and the US. In the Netherlands, the annual income gains of working-class households did not exceed 0.5% over the last decades and they were even negative in Switzerland, lagging behind the income evolution of the two middle-class segments. In Denmark and especially Finland, income gains were larger for all classes, but they followed the same linear pattern, with the lowest gains for the low-skilled working class and largest gains for the middle and upper-middle class. The evolution was different in Ireland where working-class incomes benefitted disproportionately from the Celtic Tiger boom years, although the middle and upper-middle classes also experienced strong income growth of 1.4% and more annually. Finally, Austria is the only country in our study that shows the pattern expected by the squeezed middle-class thesis. In Austria, disposable incomes only grew at the extremes – in the low-skilled working class and upper-middle class – but stagnated for the skilled working class and the middle class.

## Discussion and Conclusion

Over the past decade, a common view in the public debate was that technology has “wrecked the middle class” (Autor & Dorn, 2013b) and “hollowed [it] out in terms of wages and employment” (Jaimovich, 2020, p. 4). Our paper challenges this view with three main findings.

*First, the middle class experienced gains in both employment and income:* There has been no squeeze on the middle class in recent decades, either in terms of employment or income. In the 1980s, the middle and upper-middle classes were still vastly outnumbered by the skilled and low-skilled working classes in the six large countries studied. Yet over the last four decades, job opportunities for managers, professionals, and technicians have expanded, while they have declined for laborers, assemblers, craft workers, and routine clerks. The share of the upper-middle and middle classes in total employment increased by 10–20 percentage points, while the share of the skilled and low-skilled working classes decreased by the same amount. Thus, we observe an upgrading of the class structure in Europe and the United States, mirroring the upgrading of the occupational structure found in recent comparative studies (Fernández-Macías & Hurley, 2016; Haslberger, 2021; Oesch, 2013; Oesch & Piccitto, 2019).

The middle class has not only become larger, but also held its ground in terms of income growth. Middle and upper-middle-class households fared best in Poland with annual increases of 2.5% and more. In Germany, Spain and the US, their household disposable incomes increased by about 1% per year. Only in France and the UK did the middle class tread water with annual gains of half a percent. Annual gains of 1% as in the US and Germany may seem modest when compared to the postwar decades, but they add up to income gains of 33% over thirty years, making the children’s generation one-third richer than the parents’ generation. Indeed, our cohort analysis suggests that the promise of doing better than one’s parents and grandparents held for the members of the middle and, even more so, upper-middle classes everywhere except for Generation X in the UK and possibly France.

*Second, the working class lost out:* In terms of employment, the big loser in recent decades has been the working class. In the wake of skill-biased technological change, globalization, and the neoliberal turn in politics, the labor market opportunities of the working class have deteriorated. The employment share of the skilled and low-skilled working class fell in all the large and small countries studied. As a result, the working class is losing the majority status that it enjoyed in Europa and the US for most of the 20th century.

In terms of disposable income, working-class households fared comparatively well in the UK after 1998, with annual gains of one half to 1%, and in Spain with over 1%. Yet they fared best, by far, in Poland with annual rises of 4–5% per year in the first two decades of the 21st century. By contrast, in France, Germany, and the US, the working classes experienced only minimal rises, with less than half a percent annually. Income trajectories across cohorts were particularly bleak in the US and Germany. In the US, the march towards economic prosperity had stalled for the working-class household of the Baby Boomers and Generation X. Worse still, in Germany, the working-class cohorts that followed the Silent Generation saw their living standards fall.

*Third, cross-country differences loom large:* Our analysis reveals large country differences. Spain and particularly Poland stand out as having experienced much larger income gains than the other four countries. Starting from lower levels of economic prosperity, these two countries were further away from the world’s technology frontier and benefitted from catch-up GDP growth. Between the second half of the 1990s and the Great Recession of 2008, Spain’s economic boom was driven by strong domestic demand and a construction boom, leading to massive job creation across the skill spectrum. In parallel, labor force participation of working-age women increased dramatically in Spain, from initially low 42% in 1990 to 70% in 2020 (OECD statistics). Spanish households were thus able to increase their incomes not only because hourly earnings increased, but also because more people per household were in paid employment.

Catch-up growth was even more impressive in Poland where GDP per capita was only 32% of the U.S. level in 2000, but reached 53% by 2020 (OECD statistics). A major driver of Poland’s growth was manufacturing. Thanks to lower wage costs and the adhesion to Europe’s single market in 2004, Poland became the extended workbench of Western Europe, especially Germany. While the other large countries experienced steady deindustrialization in recent decades, Poland’s industrial sector continued to employ more than 30% of the workforce in 2020 – compared with less than 20% in France, the UK and the US.

However, our results for Poland are sensitive to period effects. Income inequality in Poland had spiked in the 1990s after the country’s abrupt transition from a communist to a market economy, with working-class households suffering disproportionately from the shock therapy’s high unemployment and stagnant incomes (Bukowski & Novokmet, 2021, p. 189). Had we been able to cover the 1990s, our results would have shown slower income growth for Poland, especially for the working class.

In contrast to Poland and Spain, the turn of the century in Germany was marked by crisis. In the 1990s, the post-reunification recession led to rising unemployment that weakened trade unions, works councils, and collective bargaining. Mass unemployment and weaker unions paved the way for labor market deregulation in the form of the Hartz laws (Baccaro & Höpner, 2022; Carlin & Soskice, 2008). The combined result was falling employment, weaker bargaining power and stagnating incomes for the working class. In the early 2010s, Germany left its long slump behind and embarked on sustained GDP growth. Thanks to the return to full employment and the introduction of a minimum wage in 2015, this has also improved the prospects of working-class households.

*What are the implications of decline of the working class*? Our main finding is that the working class lost out everywhere in terms of employment and, in Germany and the US, in terms of income. Much of the recent political turmoil in Western democracies has to do with working-class decline. As markets and politics failed to deliver improvements in living standards, growing sections of the working class turned towards candidates and parties of the radical right. In a context of shrinking job opportunities and stagnating incomes, these parties’ angry opposition to globalization, multiculturalism, and national elites struck a chord with disaffected working-class voters (Bornschier & Kriesi, 2012; Hall et al., 2023).

Given the empirical evidence, we see only one way to save the thesis of a middle-class squeeze: by arguing that there is no such thing as a working class, because the middle class begins where poverty ends (Ravaillon, 2010). This semantic argument has been adopted by many economists and international organizations such as the OECD. But it is so clearly at odds with the recent history of industrial societies that it requires a healthy dose of amnesia. Moreover, it ignores survey evidence showing that even in the early 21st century, sizeable swathes of citizens consider themselves to be working class (Oesch & Vigna, 2023).

A final question remains: Why has the narrative of a middle-class squeeze gained so much traction despite the lack of evidence? In addition to the argument that the middle class has replaced the working class in the language of the 21st century, two other arguments focus on morality and expectations. According to a moral argument, the stagnation of working-class incomes may not have been overly troubling to many pundits. It seemed only natural that in the knowledge economy, workers with little education would see their incomes stagnate. The perception of the problem changed, however, when white-collar workers with postsecondary degrees began to see their income growth slow. For the educated middle class, the stalled economic elevator seemed entirely undeserved and a broken promise.

Finally, the middle-class squeeze thesis may also stem from people’s expectations of income growth. Three decades of massive GDP growth after 1945 led to firmly entrenched expectations of rising incomes and living standards. Workers socialized in this context came to view annual income gains of 1% as a step backward (Inglehart & Norris, 2017). Moreover, the slowdown in economic growth meant not only that there was less income to distribute – but, crucially, that this income was distributed unequally as a small elite class pocketed the lion’s share in the new Gilded Age (Hacker & Pierson, 2010; Piketty, 2014). So the claim that the middle class has been left behind is true when compared to the fortune of those *at the top*. However, it completely ignores the fact that in most countries the real losers in recent decades were situated *at the bottom* – the working class.

## Supplemental Material

Supplemental Material - The Myth of the Middle Class Squeeze: Employment and Income by Class in Six Western Countries, 1980–2020 Supplemental Material for The Myth of the Middle Class Squeeze: Employment and Income by Class in Six Western Countries, 1980–2020 by Jad Moawad and Daniel Oesch in Comparative Political Studies

## Data Availability

The data used in this article are provided by the Luxembourg Income Study LIS (https://www.lisdatacenter.org/). The replication materials and R codes of all our analyses are available in \Moawad and Oesch (2024).

## Notes

1. For the US: *New York Times,* “What’s really squeezing the middle class?”, April 25 2007. *Financial Times*, “The crisis of middle class America”, July 30 2010. *Wall Street Journal*, “The middle class squeeze”, September 25 2015. For the UK: *Guardian*, “Robots will not lead to fewer jobs – but the hollowing out of the middle class”, August 20 2017. For Germany: *Spiegel,* “Deutschlands Mittelschicht schrumpft dramatisch“ [Germany’s middle class shrinks dramatically], December 13 2012. *Süddeutsche Zeitung*, „Schrumpfende Mittelschicht: arbeite hart, aber besser geht es dir nicht“ [Shrinking middle class: work hard, but you won’t fare any better], August 29 2015. For Spain: *El Diario* “Adiós a la clase media” [Farewell to the middle class], December 18 2014. *El País*: “La Clase media menguante” [The declining middle class], September 29 2019.

2. If we restrict our analysis for Germany between 1984 and 2018 to households residing in West Germany, we find almost exactly the same pattern of income growth by class.

3. “Only a few broad ‘skill level’ categories can usefully be identified for cross-national comparisons. ISCO-88 uses four skill levels to define the broad structure of the classification at its most aggregate level” (Elias & Birch, 1994, p. 2).

4. Our decision to include information on education is motivated by both practical and theoretical reasons. Practically, as our data only distinguish occupations at the ISCO 1-digit level, the use of education avoids misclassifying as upper-middle class managers with only compulsory education (e.g., lower grade managers of small shops) or professionals without higher education (e.g., nurses or pre-school teachers). More fundamentally, education and occupations are systematically linked, as access to many occupations depends on educational qualifications. Many professions, such as doctors and lawyers, require a university degree and individuals cannot enter these professions without higher education. Note, however, that we have replicated all our analyses without information on education and results lead to the same conclusions.
