# Supplementary material for: The Myth of the Middle Class Squeeze: Employment and Income by Class in Six Western Countries, 1980–2020
Source: Comp Polit Stud. 2024 Aug 8;58(8):1636–69. doi: 10.1177/00104140241271166 (PMC12083865; doi:10.1177/00104140241271166)
Supplement: Supplemental Material - The Myth of the Middle Class Squeeze: Employment and Income by Class in Six Western Countries, 1980–2020 [file sj-pdf-1-cps-10.1177_00104140241271166.pdf]

Appendix A – supplementary materials

Figure A.1: Annual change in household disposable income (upper panel) and household labor income (lower panel) based on dominant household class, in %

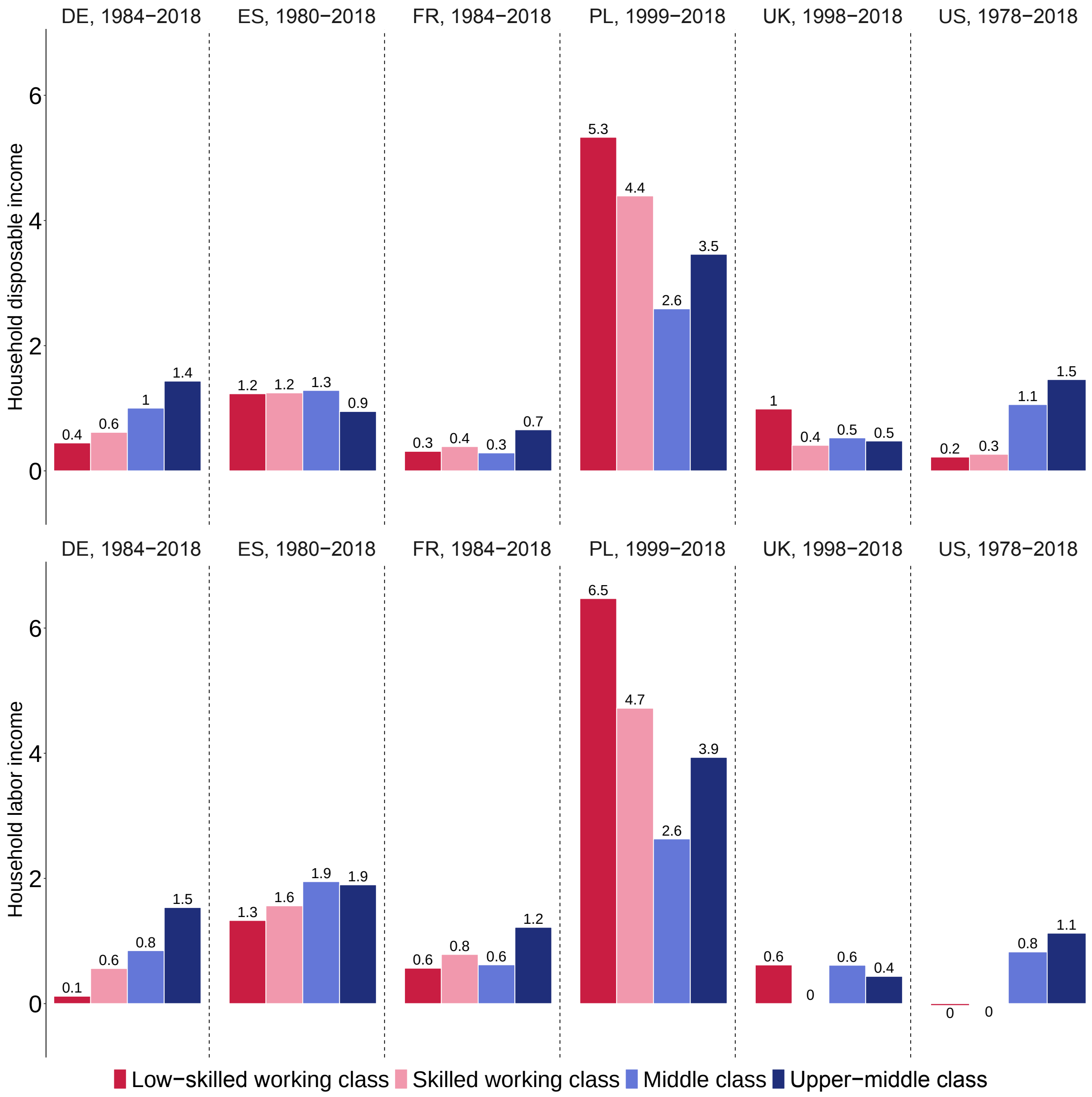

Figure A.2: Annual mean change in household disposable income by income quartile, in %

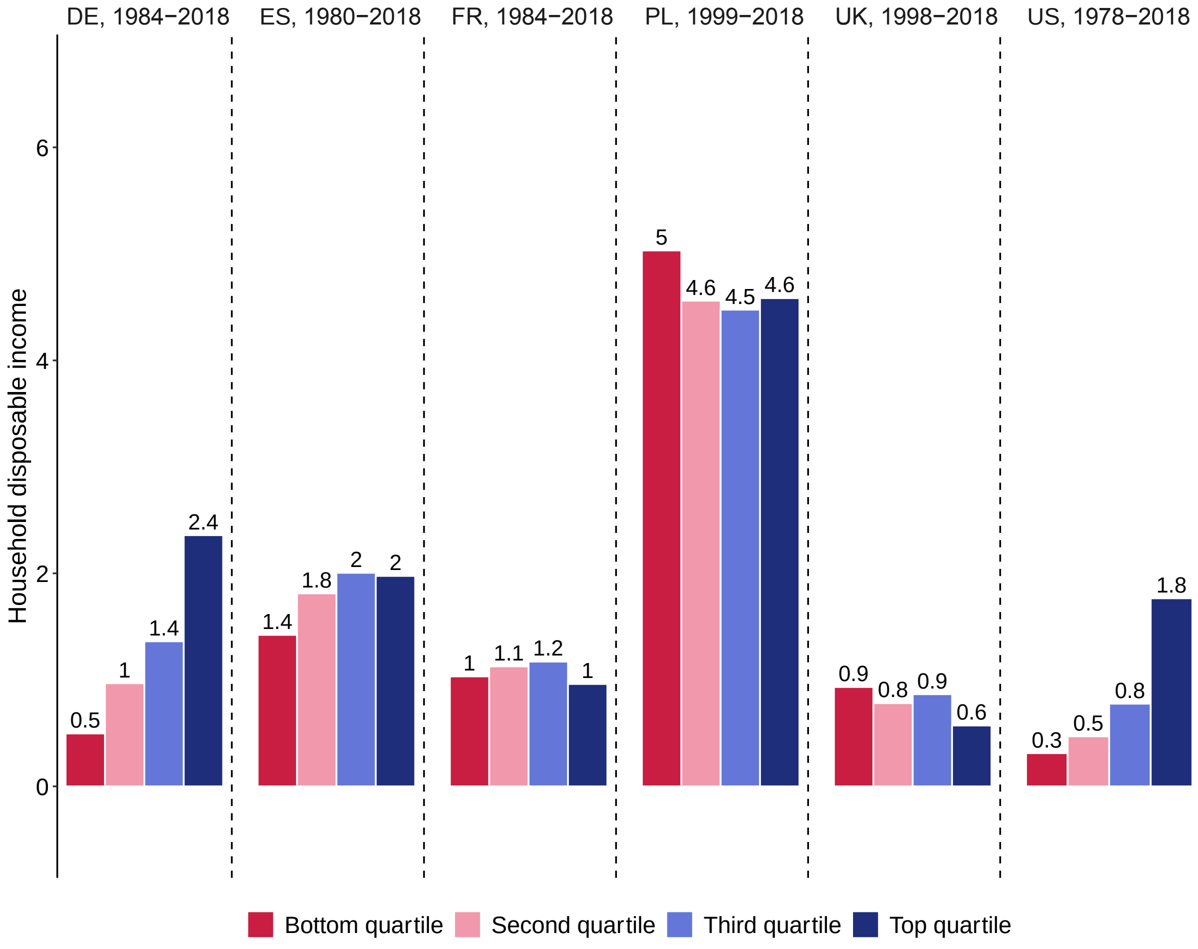

Figure A.3: Annual mean change in household disposable income by class and decade, in %

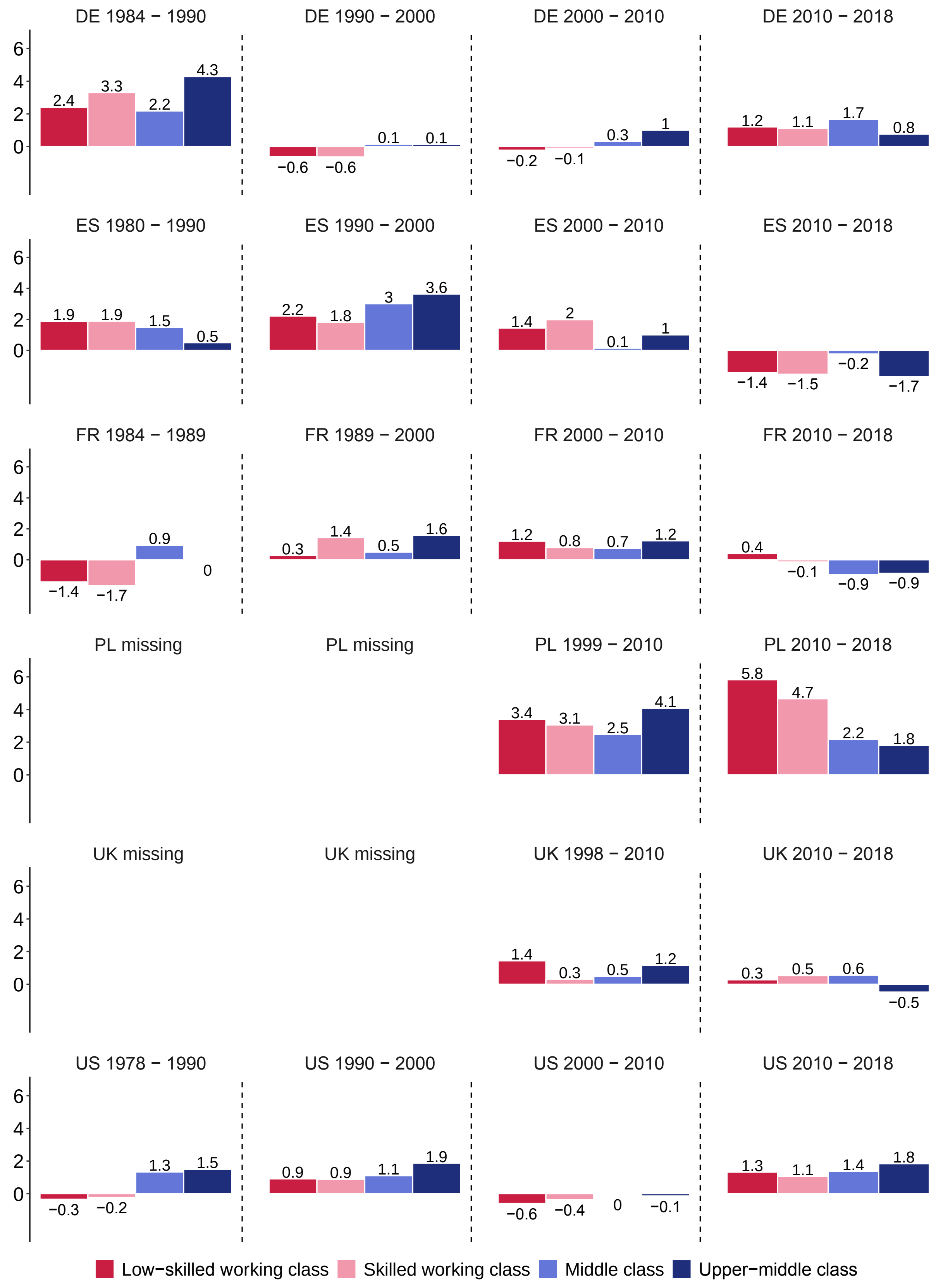

Figure A.4: Employment change by class over a common 20-year period (in percentage points)

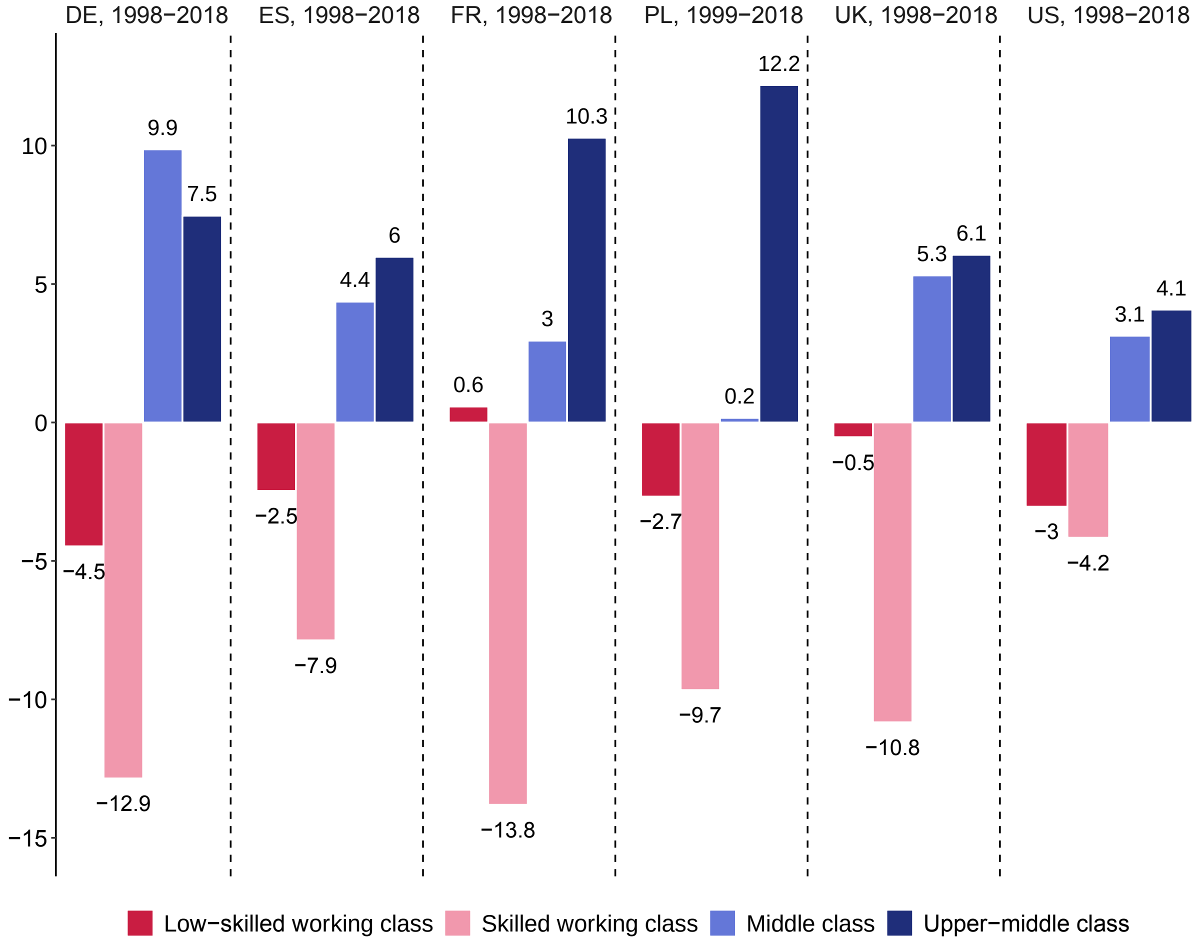

Figure A.5: Annual mean change in household disposable income by class over a common 20-year period, in %

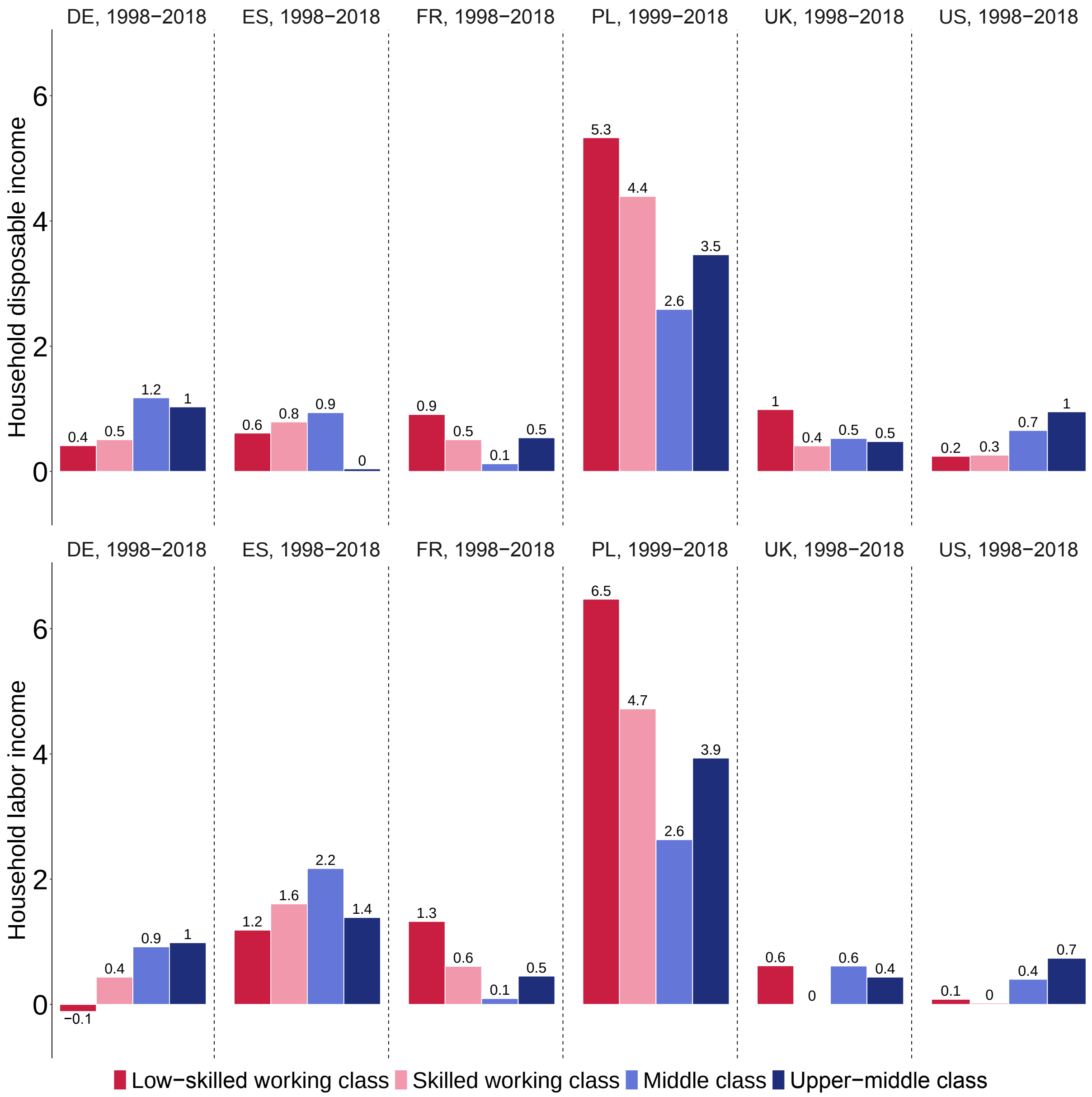

Figure A.6: Employment change by class in small and affluent European countries (in percentage points)

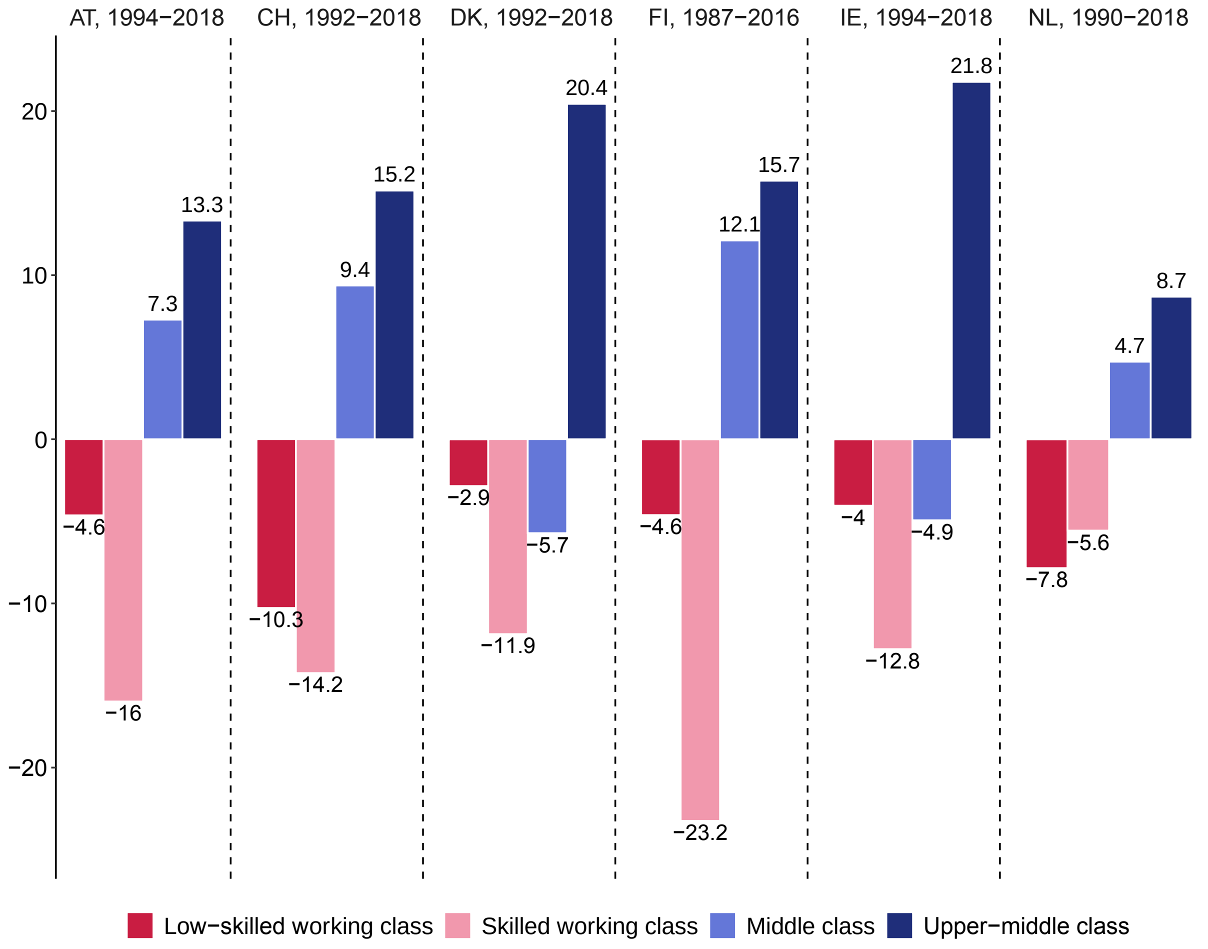

Note: AT: Austria, CH: Switzerland, DK: Denmark, FI: Finland, IE: Ireland, NL: Netherlands

Table A.1: Coding of the four-class variable with LIS-data

Upper-middle class	ISCO == 1 & status == "employer"<br>ISCO == 1 & status == "employee" & EDUC == "at least upper-secondary or higher"<br>ISCO == 2 & EDUC == "tertiary"
Middle class	ISCO == 1 & "self-employed"<br>ISCO == 1 & status == "employee" & EDUC == "less than upper-secondary"<br>ISCO == 2 & EDUC== "less than tertiary"<br>ISCO ==3<br>ISCO == 4 & EDUC == "tertiary"<br>ISCO == 4-8 & status == "employer"
Skilled working class	ISCO == 4 & status == "employee" or "self-employed" & EDUC == "less than tertiary"<br>ISCO == 5,7,10 & status == "employee"<br>ISCO == 8 & status == "employee" & EDUC == "upper-secondary or higher"<br>ISCO == 5-8 & status == "self-employed"
Low-skilled working class	ISCO == 9<br>ISCO == 6 & status == "employee"<br>ISCO == 8 & status == "employee" & EUDC == "no upper-secondary"

Table A.2: Descriptive statistics

			DE		ES		FR		PL		UK		USA	
			1984	2018	1980	2018	1984	2018	1999	2018	1998	2018	1978	2018
Household disposable income		Mean	22,788€	31,416 €	11,962 €	20,010 €	21,579 €	28,571 €	20,824zł	39,396zł	24,114 £	27,905	36,918 $	53,687$
Size of classes	Low-skilled working	%	25	14	32	22	31	19	24	21	16	15	23	15
	Skilled working class	%	43	27	48	37	40	28	52	44	43	34	40	31
	Middle class	%	19	36	13	22	19	27	14	14	16	21	14	21
	Upper-middle class	%	13	23	8	19	10	26	10	21	25	30	23	33
Household size		Mean	3.2	2.9	4.2	3.1	3.2	2.8	3.7	3.3	2.8	2.8	3.3	3.1
Houshold head	Male	%	88	52	94	64	88	79	70	70	85	60	83	52
Age		Mean	41	45	42	44	40	43	41	42	41	42	40	42
N observations			6,964	19,321	34,815	17,916	12,912	45,378	42,411	40,724	21,519	17,182	63,362	78,279

Table A.3: correspondence of classes in the European Socio-Economic Classification (ESeC) to the four-class measures

ESeC classes (EGP terms in parentheses)	*This paper's classes*
1 Upper salariat (or higher-grade service class)	Upper-middle class
2 Lower salariat (or lower-grade service class)<br>4 Petite bourgeoisie with employees<br>5 farmers with employees	Middle class
3 Higher white collar & 6 higher blue collar<br>4 Petite bourgeoisie without employees<br>5 farmers without employees<br>8 Skilled manual	Skilled working class
7 Lower grade white collar<br>9 Semi-/skilled workers	Low-skilled working class

Appendix B – additional analyses with a 5-class schema

Table B.1: Coding of the five-class variable with LIS-data

Upper-middle class	ISCO == 1 & status == "employer"<br>ISCO == 1 & status == "employee" & EDUC == "at least upper-secondary or higher"<br>ISCO == 2 & EDUC == "tertiary"
Middle class	ISCO == 1 & "self-employed"<br>ISCO == 1 & status == "employee" & EDUC == "less than upper-secondary"<br>ISCO == 2 & EDUC== "less than tertiary"<br>ISCO ==3
Lower-middle class	ISCO==4<br>ISCO == 4-8 & status == "employer"<br>ISCO == 4-8 & status == "self-employed"
Skilled working class	ISCO == 5,7,10 & status == "employee"<br>ISCO == 8 & status == "employee" & EDUC == "upper-secondary or higher"
Low-skilled working class	ISCO == 6 & status == "employee"<br>ISCO == 8 & status == "employee" & EUDC == "no upper-secondary"<br>ISCO == 9

Figure B.1: the class composition of the workforce over time (in %)

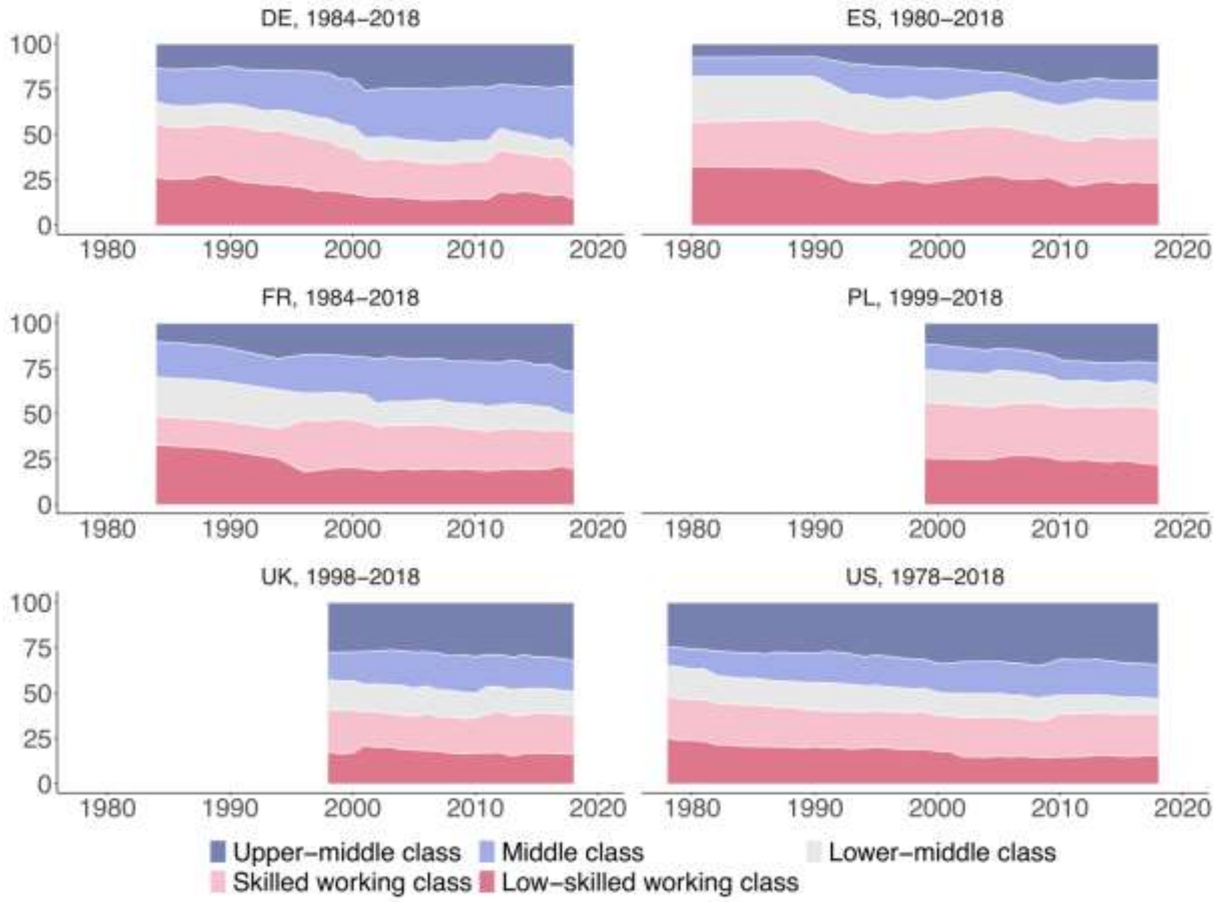

Figure B.2: change in the employment share of different classes (in percentage points)

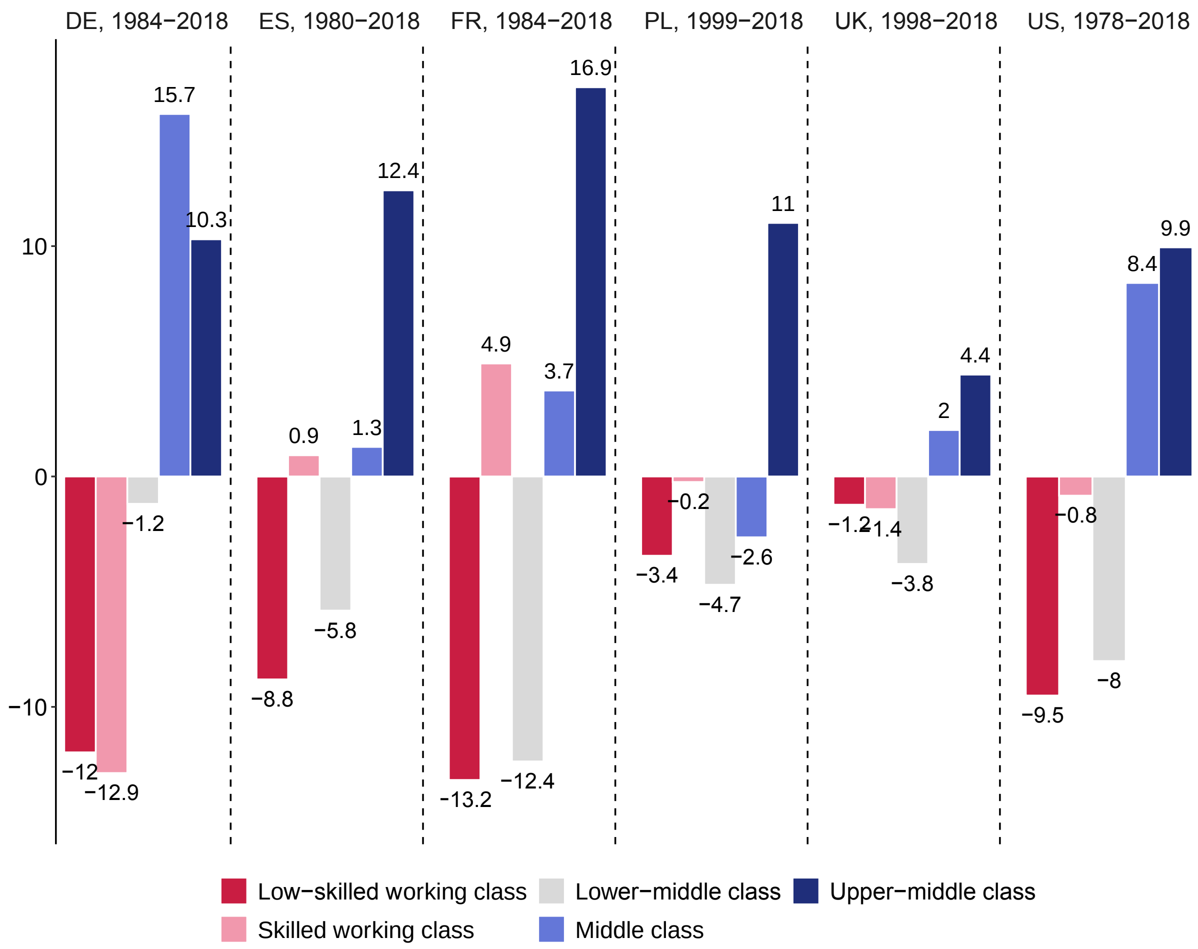

Figure B.3: evolution of indexed real household labor income by social class over four decades

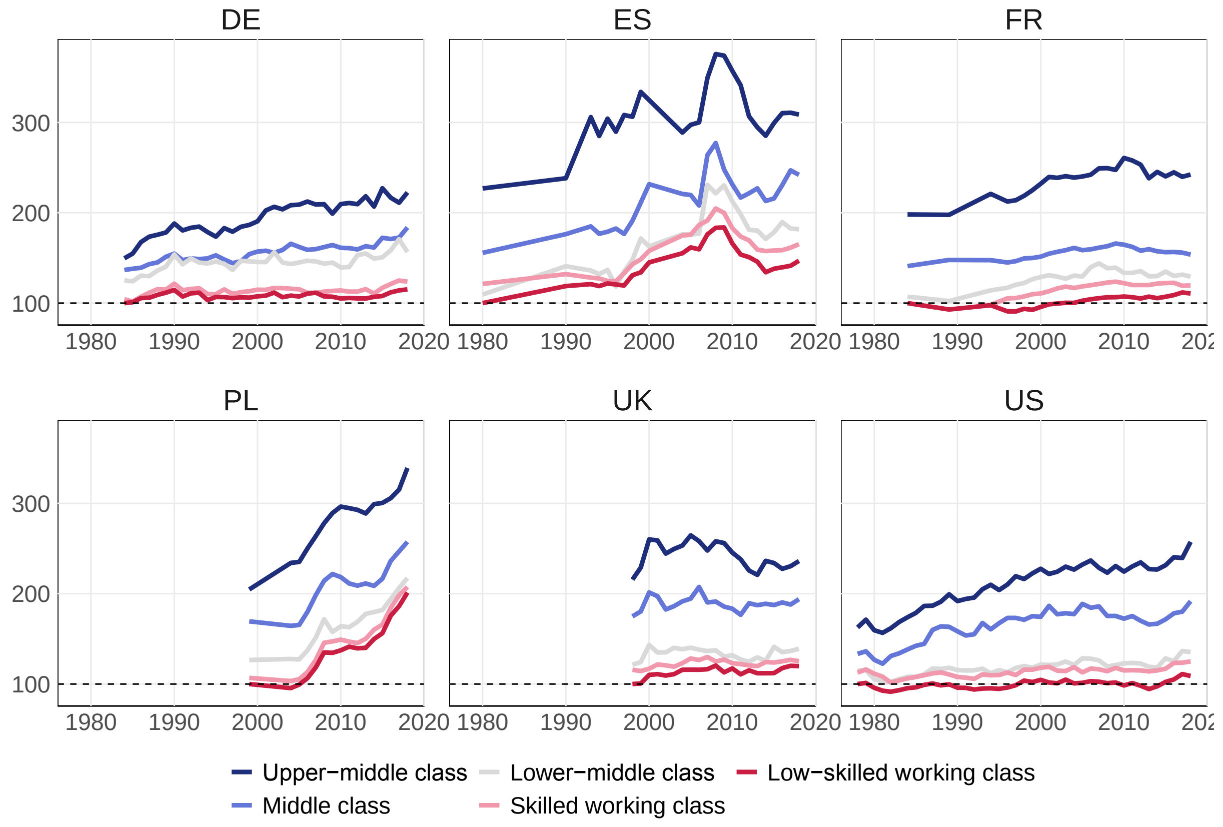

Note: household labor income is corrected for inflation and adjusted for household size. Values are indexed for the low-skilled working class in the first year of observation (that is, all incomes are expressed relative to the income of the low-skilled working class which is set, within each country, at 100 at the beginning of the time series).
